# Video Q&A: Tobacco-related mortality: past, present and future. An interview with Alan Lopez

**DOI:** 10.1186/s12916-014-0162-x

**Published:** 2014-10-21

**Authors:** Alan D Lopez

**Affiliations:** The University of Melbourne, Building 379, 207 Bouverie St, Carlton, 3053 VIC Australia

**Keywords:** Tobacco, Smoking, Mortality, Disease burden, Global health, Epidemiology

## Abstract

In this video Q & A, we talk to Alan Lopez about the global burden of tobacco-related mortality. Prof Lopez highlights how this epidemic emerged in the past, and how it is likely to progress in the future. He also discusses ways in which countries that currently have few or no tobacco-control policies in place can apply measures to curtail premature mortality.

**Electronic supplementary material:**

The online version of this article (doi:10.1186/s12916-014-0162-x) contains supplementary material, which is available to authorized users.

## Introduction

**Figure 1:**
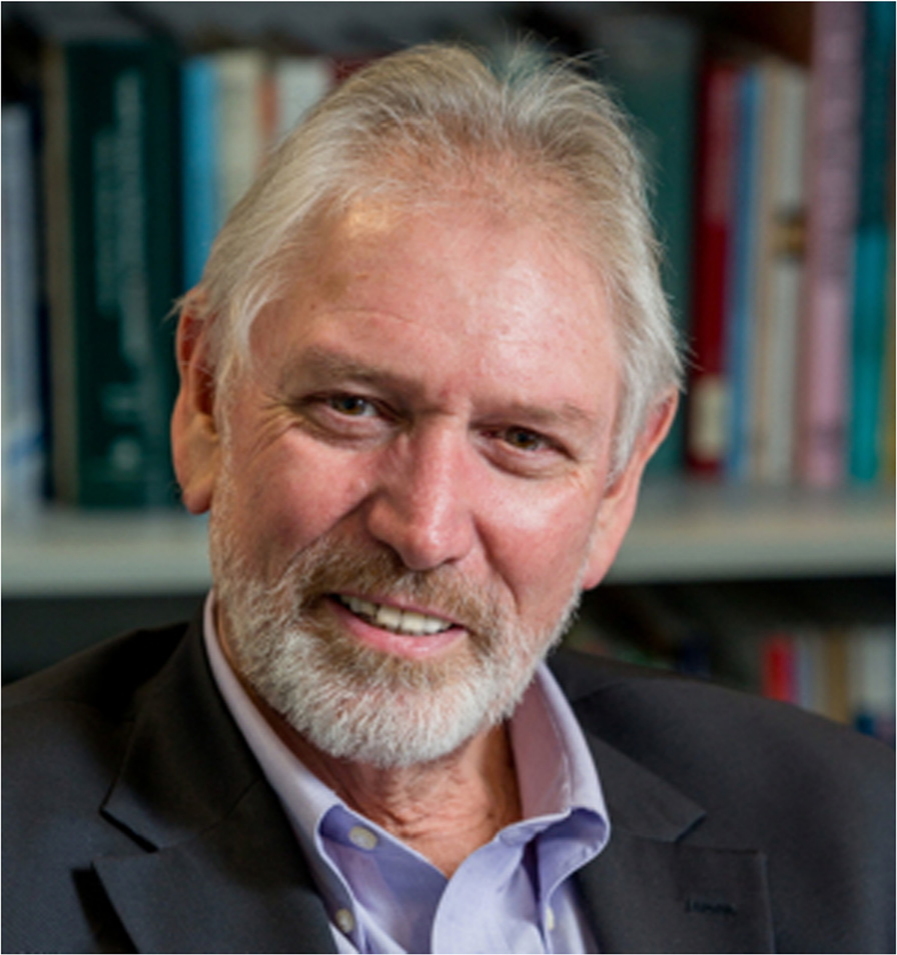
**Professor Alan Lopez.**

Professor Alan Lopez (Figure 1) is currently Melbourne Laureate Professor and the Rowden-White Chair of Global Health and Burden of Disease Measurement at The University of Melbourne. He is also Director of the Global Burden of Disease Group in the Melbourne School of Population and Global Health.

Prior to his current role, he held positions at the University of Queensland from 2003 to 2012 as Professor of Medical Statistics and Population Health, Professor of Global Health, and Head of the School of Population Health. Before this, he spent 22 years at the World Health Organization (WHO) where he was Chief epidemiologist in WHO’s Tobacco Control Program (1992 to 1995), Manager of WHO’s Program on Substance Abuse (1996 to 1998), Director of the Epidemiology and Burden of Disease Unit (1999 to 2001) and Senior Science Advisor to the Director-General (2002).

He has published seminal research on the Global Burden of Disease. and with Sir Richard Peto, developed the *Peto–Lopez* method, which is widely used to estimate tobacco-attributable mortality, and has been used to support policy action. In this interview (Additional file 1), we talk to Prof Lopez about his work on tobacco-related mortality, and ways in countries can apply tobacco control measures to curtail premature mortality.

## Edited transcript

### 1) You have published seminal work with regard to tobacco-attributable mortality. What led you to suspect that tobacco has a large impact on life expectancy?

The work on tobacco mortality came out of work that I was doing when I was a PhD student. I was looking at mortality rates historically in Australia, and noticed how they had changed dramatically, particularly for men, and I wanted to know what was behind this change. The sex differential in mortality, which is what I was studying, was about three years at the turn of the century (similar to the UK and the US) and rose to about eight years in the mid-1970s. A lot of this had been linked to tobacco, but it was very difficult to actually pinpoint the impact of tobacco from what I call ‘national mortality statistics’. But I knew it was there, and I got very, very interested in trying to explain these big divergences in sex mortality differentials, looking at the impact of tobacco.

### 2) What stimulated your specific interest in tobacco-attributable mortality as compared to general mortality trends?

As a PhD student, I was looking at all this mortality data available – and felt there’s a story here – something’s driving this. However, I was not a qualified or capable epidemiologist – I was just a student at that stage. But I had developed a strong interest in not just describing health trends and mortality, but also in the causes of death. I also wanted to know what were the ‘causes’ of those causes of death? What was driving the lung cancer rate in Australia and comparator countries like the UK? What was driving the rise in ischemic heart disease and stroke, and so on? So I started to read a lot about epidemiology and the epidemiology of non-communicable diseases. That’s how I first came into contact with the work of Richard Doll and Richard Peto, and read what they had done.

They’d done a lot of analytical studies, particularly in the UK with the British doctors who they’d been following, at that stage, for about 20 years from a cohort in the early 1950s. I started to look at their work, and was very impressed by the quantification of the impact of tobacco when you looked at it in defined populations – in this case, the British doctors. But the Americans were doing the same thing; the American Cancer Society had set up a large cohort, and they were looking at the causes of individual deaths and relating them back to what these individuals had smoked and what they ate and other things. They were coming up with very, very significant risks for various diseases caused by tobacco – lung cancer, chronic lung disease – and I was very impressed by this. At the time, I was not doing these kinds of studies, but I knew that the explanation to the findings of research that I was doing lay in this kind of epidemiology. So I got extremely interested in not only the broad descriptive trends, but what was causing those trends.

### 3) What were the trends of tobacco-attributable mortality 20 years ago as compared to today?

Twenty years ago (that is, the early 1990s), we were seeing several things happening. When you talk about trends, you need to disaggregate the exposure group. So, firstly, let’s talk about males and females. Women really only started to smoke in countries like the UK, Australia and the US after the First World War, perhaps a long time after the First World War – the 1930s, 1940s. So they were not yet dying in large numbers in the late 1980s and early 1990s.

Men, on the other hand, in the UK, Australia and the US, had been smoking for up to seven decades ,and so their epidemic was what we call ‘quite mature’. They were beginning to die in large numbers in the 1960s and 70s, and by the 1990s something like one-third to 40% of deaths in middle-aged men were due to tobacco. That’s a massive premature mortality, and reflects the long delay between populations taking up smoking in large numbers and then dying from it, also in large numbers. In the 1990s, middle-aged women in the UK and Australia were just beginning to die of tobacco-related causes – however, not at the same level as men because they had always smoked less than men. As women smoked later than men, we were in this very interesting position where some countries were beginning to see rapid rises in female smoking-attributable mortality, but not yet in other countries such as France.

In the 1990s, you could go to countries, like Spain, Portugal, France, and you would see women smoking everywhere! But they were young women who were smoking. Their mothers – the older women – did not smoke, and so at the time there was very, very little tobacco-attributable mortality among women in these countries. But we’re beginning to see that now in French, Spanish, and other southern European women because they’ve now been smoking long enough to begin to kill themselves in large numbers.

In developing countries, on the other hand, we didn’t really see the impact of tobacco in populations. Richard Peto and I estimated that it was killing about one million people in developing countries: a few hundred thousand in China, a few hundred thousand in India, a few hundred thousand, perhaps, in Latin America. But while these numbers may sound large, they’re much, much less than what we’re going to see. So the epidemic was at different stages in different population groups – males and females, developed and developing countries.

### 4) How do you think the global tobacco-attributed mortality rates will change in the future?

We’ve seen enough to understand the evolution of the epidemic. Let’s take developing countries, if I can use them as a whole. One in two men in developing countries smoke cigarettes every day. But they’ve only really been smoking in China and populations like that since the 1970s or so, which means we’re not yet seeing the full effects. In fact, we’re not even seeing much of the effects of tobacco on these populations of males. But we will. In the last two decades, they’ve continued to smoke in large numbers.

In fact, smoking prevalence has not declined at all – marginally in China, but it’s very hard to see significant changes. Still, six out of ten Chinese men smoke; six out of ten Indonesian men smoke; three or four out of ten Indian men smoke. These are very, very large populations, and if they don’t stop smoking – and it’s very hard to see that that will happen without concerted global tobacco control action – then we’ll see large numbers continuing to smoke, something like what we saw in the US and the UK, and eventually the same amount of tobacco-attributable mortality in these populations. But it’s still too early to see that.

Women, on the other hand, have not taken up smoking, by and large, in developing countries. Still less than 10%, on average, of women in these populations smoke. They’re a huge target for the tobacco industry, but we don’t yet see great amounts of smoking, so we’re not predicting large amounts of tobacco mortality in women in those countries. We will see it in men, though.

Developed countries over the last two decades, I think, have been characterised by some fantastic work by tobacco control advocates. Whatever country you look at – particularly the Anglo-Saxon countries (Australia, UK, US), the effects of tobacco control measures have been real, and we’ve seen massive declines in smoking consumption and prevalence. In Australia after the Second World War, something like 70% of men smoked. It was the same in the UK. Now 15 to 18% of men smoke. So there have been huge declines as a result of tobacco control measures. A lot of those have taken place in that period since 1990, or at least have consolidated. We have not seen, really, any increases in prevalence during that period.

### 5) If tobacco control measures in many developed countries have been successful, how can these go on to inform further interventions, particularly in countries where interventions to combat smoking are rare or do not exist?

What I would suggest is that they draw on the experience, the lessons learned, from countries where control measures have worked. Smoking control ***can*** work. We have seen huge declines in prevalence. We’ve seen huge declines in consumption. On average, per capita smoking in the UK and Australia was probably 4,000 cigarettes per adult per year in the 1960s, prior to the US Surgeon General’s report; it’s now about 1,000. So there’s been a 75% decline in the amount of tobacco consumed. It can work. It doesn’t mean the problem is over, but we’ve seen huge declines. I think the lesson for developing countries is: why did that happen and how can we translate that knowledge to help curb our epidemics?

There are relatively straightforward things that can be done. It’s difficult to evaluate formally the actual impact of A versus B versus C as an intervention. But what we can see is that the cocktail of interventions – which has to include price rises, that is, increasing taxation, is a sure way to cut consumption. There needs to be strict controls on advertising and promotion. Look at the UK and Australia – you can’t smoke indoors in public places. Increasingly, it’s very limited where you can smoke. That kind of public response to limiting the promotion of tobacco, the advertising of tobacco, the facility with which tobacco can be purchased – that is, in vending machines – or consumed – that is, in public places – all of those restrictions together, those interventions, have led to what we see: the huge decline in tobacco consumption.

It is really up to developing countries to say, *“This is nonsense. We cannot have one in three, one in two of our population dying from tobacco. We need to do something about it.”*

### 6) What specific cultural considerations should be accounted for when planning anti-smoking interventions?

Anywhere where you plan on implementing an intervention, you need to take into account the likelihood of it working. I think for these [low- and middle-income] countries to just pick up the legislation or pick up the advertising controls that other countries like the US or the UK have used, would be a mistake. They absolutely need to consider how these interventions can be most effective when implemented in their population. That may mean a number of ways of implementing. It may mean using cultural figures or religious figures who have more impact in those populations than they might have in the West. I think these cultural considerations need to be carefully thought through.

The tobacco industry are right on to that. They know exactly what it is in these cultures that will press the buttons to get people to smoke. They’re using them already, and certainly, tobacco control has to be as intelligent as the industry in respecting, but exploiting cultural considerations.

### 7) Are there any differences in tobacco-attributable mortality/morbidity between high- and low-income countries?

There are a number of differences. Firstly the volume, as I mentioned earlier. Tobacco currently kills about six million people a year. I know that sounds ridiculous, but there would be six million more people alive today, every year, had tobacco never been discovered. So it kills a lot of people, but it’s going to kill a lot more people.

Sir Richard Peto and I quantified the amount of death from tobacco in the 20th century, and we estimated that tobacco last century killed about a hundred million people- mostly in developed countries. In this century, the 21st century, tobacco is going to kill about a ***billion*** people, mostly in developing countries. So the numbers are quite different. The timing of the epidemics are quite different. But also the way that tobacco kills people in developing countries is different to the way that it kills people in developed countries. What tobacco tends to do is take large existing hazards in the population and multiply them up.

In the UK, Australia and the US, those hazards are mostly around vascular disease. It’s not unimportant as a cause of cancer, but tobacco kills most people in developed countries through vascular disease because that’s the biggest background risk. In developing countries, for example in China, where the biggest background risk is chronic obstructive lung disease (COPD, that is, chronic bronchitis and emphysema) what tobacco does is to take that risk and multiply it up. So, from the large prospective studies in developing populations that Richard Peto and others have done, we can see it’s killing people more from COPD and cancer than it does from vascular disease. It doesn’t mean that it won’t kill them in large numbers from vascular disease, but at the moment it’s taking other hazards and multiplying them up.

### 8) How have your findings influenced policy?

It’s always hard to know. One has to be reasonably modest about the difference between creating science and seeing it implemented. But I do think there have been some reactions, and reactions in a constructive way. When I was Chief Epidemiologist in WHO’s Tobacco Control Program in the early 1990s, I was very dismayed to see the way that the tobacco industry took what WHO said – which ought to be a global authority – and said*, “Well, one day WHO says this number of people die from tobacco, the other day it says some other number!”* This greatly weakened WHO’s effectiveness, in my view, as being a global leader in tobacco control.

So one of the first things I did was begin to work with people like Sir Richard Peto on getting the numbers right and assessing – how much do we know and how much don’t we know? And what can and cannot be reliably said about the amount of disease burden attributable to tobacco?

Having spent quite a bit of time with Sir Richard and colleagues doing that, I felt that WHO was in a much stronger position to base its evidence and advocacy work, because it had spent quite a lot of time trying to get the numbers right. If you don’t have confidence in the numbers, then your policy and advocacy work is going to be attacked by the tobacco industry, which it was.

That hasn’t happened, or at least it didn’t happen subsequently, as a result of that effort. I’d like to think that just trying to get the science right, the epidemiology right, was of great help both to national tobacco control efforts, but also to global efforts like those being advocated by WHO. Indeed, I think the work that Richard Peto and I did, and then, more recently, that Chris Murray and I did, on the burden of disease attributable to tobacco compared to other risks has helped. The great advantage of the burden of disease work is that it allows you to compare tobacco burden with blood pressure burden, with occupation and so on, so that you can get a better appreciation of just how much death and disease burden it is causing.

That work, I think, has been instrumental in getting WHO to take tobacco control more seriously. When Gro Harlem Brundtland became Director General of WHO in 1998, she was very serious about evidence and said*, “There’s a massive amount of evidence that tobacco is causing a massive amount of health loss and will cause a lot more in our lifetime, what should WHO do about it?”* I think that the epidemiology work we did, though not the only cause, was certainly one of the drivers for WHO creating its Tobacco Control Initiative and taking tobacco a lot more seriously – working on the Framework Convention, leading that Framework Convention, being much more of a beacon for countries to look to and say – what should we do and how effective will it be?

### 9) In terms of developing countries, have there been any large-scale cessation strategies and is there any evidence that these are working?

I think it’s early days. In countries like China, for example, Judith Mackay and others have been working for decades on tobacco reduction. It’s only now that governments in these countries are beginning to understand the tsunami of tobacco-related mortality that’s coming down, so they’re beginning to take some measures. For example, China is beginning to restrict advertising, although it’s not yet working successfully on taxation, but we’re going to keep at it. So, we’re beginning to see some changes. Prevalence is beginning to come down, but falls have been modest. There’s a long way to go. Going from 65% prevalence of men to 60% is a small victory, but it’s an initial victory on a long road.

In many other populations, we don’t see a lot of reduction in tobacco use. In Indonesia – another large population – there has been very, very little change in tobacco use. We see different trends in Latin America – large populations like Brazil and Mexico do not seem to be reaching the same levels of prevalence that we saw in the West and that we see in parts of Asia, like China and Indonesia in particular.

There is evidence that you can avoid large tobacco consumption in populations, through interventions. But there’s not a lot of evidence yet in developing countries that those interventions are bringing down prevalence very much once it’s reached a high level.

### 10) How do you think tobacco-related diseases will change in the next twenty years?

They will increase - unless there’s a massive amount of cessation among those 50% of men who smoke in developing countries. Something like 900 million – almost one billion people – smoke every day. If we’re going to see the same hazards eventually in these populations – most of which are in developing countries – as we’ve seen in rich populations, then one in two of those smokers is going to die from tobacco use. That’s why we get these very large predictions of one billion smokers dying this century from tobacco.

It’s very, very difficult to see how this will be avoided, without huge successful cessation programs. The best way to kill yourself is to start smoking before you’re 20 and then continue to do so. Then you’ve got a better than one in two chance of dying prematurely from tobacco use. That’s what we see happening wherever we study it. We see the age of initiation into tobacco, particularly for males, is getting younger and younger. So we’re getting more and more cohorts of young males in developing countries starting to smoke before they’re 20, and smoking 16–18 cigarettes per day. Large populations are wealthy enough now to be able to smoke significant amounts of tobacco, and they’re not stopping. If that’s the case, then in our lifetime – sometime in the 2020s and 2030s – we’re going to see that annual toll of tobacco rise from six million to ten million and then it’s going to go beyond that. By the middle of this century, tobacco – without any great change in cessation – is probably going to be killing 12–15 million people a year. These are absurd numbers! But this is going to happen unless we are successful in reducing consumption, particularly in men, and particularly in developing countries.

### 11) Where can I find out more?

See references [1–20].

## Additional file

Additional file 1:
**Interview with Alan Lopez on tobacco-related mortality.**
